# Successful implementation of Medical Education Faculty Development Project at Saint George University of Beirut in the immediate post triple blow to Beirut

**DOI:** 10.12688/mep.19519.1

**Published:** 2023-02-28

**Authors:** Alexandre Nehme, Rachel Btaiche, Marc Jreij, Jizel Jahjah, George Karam, Anne Belcher

**Affiliations:** 1 Faculty of Medicine, Saint George University of Beirut, Ashrafiye, N/A, Lebanon; 2 School of Education, Johns Hopkins University, Baltimore, USA

**Keywords:** Faculty Development, Medical Education, Kirkpatrick’s evaluation framework, Beirut’s triple blow, University Medical Center

## Abstract

**Background:** The aim of this study is to explore the efficacy of the Faculty Development Program (FDP) implemented at the Saint George University of Beirut-Faculty of Medicine (SGUB FM) under exceptional circumstances as the triple blow to Beirut.

**Methods:** The Faculty Development, directed towards a cohort of 35 faculty members, is composed of two major components: methodology of teaching and techniques of assessment. The Kirkpatrick’s assessment model, in combination with a specifically designed psychological questionnaire, were chosen to assess the effectiveness of the faculty development initiative.

**Results:** Results of the different questionnaires were interpreted individually, then through the lens of the psychological questionnaire. A majority of faculty (55%) were significantly affected psychologically by Beirut’s triple blow and 77% of all participants found the workshops to be of excellent quality (Kirkpatrick’s Level I). Moreover, Kirkpatrick’s level II results yielded a 76% mean percentage of correct answers to post-workshops MCQs and a significant improvement in the mean results of the self-assessment questionnaires, administered before and after each workshop. Results also show that the more a trainee is psychologically affected, the less he/she performs as evidenced by a decrease in the satisfaction rate as well as in the score of the cognitive MCQs and of the self-assessment questionnaires.

**Conclusions:** This study was able to highlight that significant learning can occur amidst exceptional circumstances like the Beirut triple blow and administration should invest in professional growth to retain its faculty.

## Introduction

On May 16, 2018, Saint George Hospital University Medical Center (SGHUMC), built in 1878, was granted government licensure and approval to build the Saint George University of Beirut (SGUB) and achieve a vision of creating an independent school of medicine (SGUB FM).

Saint George University of Beirut (SGUB FM), a recently established school of Medicine, is seeking the World Federation for Medical Education (WFME) through TEPDAD accreditation and aiming to create a school of medicine that will be acclaimed for its competitive student admission as well as its ability to offer unique integrated educational opportunities. SGHUMC physicians, who have limited formal instruction in education or curriculum development, are currently teaching using a didactic 20-year-old curriculum that never underwent a review process. Therefore, in the effort towards adopting modern methods of medical education, the nascent university’s leadership provided faculty members with opportunities to be trained with targeted faculty development initiatives in the knowledge and skills to produce a competency-based curriculum linking the education of future physicians to the healthcare needs of the population.

‘Most faculty development initiatives have been tested in different settings, whether in the United States or abroad, and have proved their efficacy in improving teaching effectiveness in medical education ^1– 7^. However, such initiatives have still not yet been studied when applied in a combination of exceptional circumstances such as the triple blow to Beirut: the collapse of the banking system since 2019, COVID-19 pandemic and on August 4, 2020, the Beirut Explosion with its devastating physical and psychological impact on SGHUMC. Those events have markedly destabilized the governance of the country, depleted its hard currency, and made it impossible, for an unforeseeable period of time, to run business as usual. Present at the eye of the storm, faculty members who have been hit, injured, and psychologically affected by the blast, and whose revenue streams and potential cash flow have been markedly diminished, still decided to stay in Lebanon trying to resume life as usual.

The aim of this study is to explore the efficacy of implementing a Curriculum Development Program at SGUB FM under tough economic and psycho-social circumstances through assisting individual teachers to deliver effective medical education and training.

## Methods

### Study Population

In its effort to matriculate the first MED I class in September 2022, SGUB FM’s Dean’s Office decided to direct one of its faculty development initiatives towards a cohort of 35 faculty members, including members of the Curriculum Committee and teachers of the MED I and MED II curriculum blocks and threads, who are also practicing physicians at the Saint George Hospital University Medical Center (SGHUMC).

### Settings and study design

This is an interventional study where the proposed pilot Faculty Development Program (FDP), is designed under the auspices of student/learner- centered classes with the following modules: 1. Introduction & Characteristics of Adult Learning, 2. Learner-centered Classes 3. Group Work 4. Interactive Lecturing 5. Project-based Learning 6. Flipped Learning 7. Assessment: Formative and Summative 8. Assessment: Rubrics 9. Team-based Learning 10. Reflections. The program is comprised of two major components: methodology of teaching and techniques of assessment. Since reflections are reported to be the heart of any learning experience ^8^, this training program was concluded by a reflection session where participants discussed the learning that took place as a result of the program and any perceived transformation in their teaching practices. The training program was interactive by design and extended over ten sessions, two hours each, spread over a period of six months. Participants were encouraged to engage in all these sessions and to fill questionnaires that assesses their learning.

The Kirkpatrick’s (1976) assessment model was adopted to evaluate the effectiveness of the faculty development initiative due to its simplicity, its assessment of a limited number of variables, the ease of its evaluation criteria, the lack of requirement to collect participants’ basic data or past performances as well as the independence of individual and environmental variables (Bates, 2004). The Kirkpatrick’s model, consisting of four levels (appendix 1), was used to evaluate outcomes of the FDP. Two levels were developed: reaction – Level 1 (appendix 2) and learning – Level 2 (Sessions 1 to 9 ‘Post workshop Multiple Choice Questions’ and ‘Retrospective Pre and Post’ questionnaires). The retrospective pre–post method (RPP) offers an alternative method to the traditional ‘pre-post design’ that usually rely on the stability of the participants standard of measurement for the dimension being assessed from one data point to the next ^9^. As the learners' perception of the dimension(s) being measured evolve, they readjust the criteria for their self-rating: the response shift bias ^10, 11^. When using the RPP method, the ratings of understanding before (referred to as the ‘retrospective pre’) and after (referred to as the ‘retrospective post’) the intervention employ the same metric because data are taken at the same point in time, i.e., at the conclusion of training, thus reducing such bias ^12^. The behavior level (level III) and the results level (level IV) are evaluated by qualitative and quantitative data (open- and closed-ended questions) with trainee faculty after three months and nine months of workshops completion respectively.

Finally, a specific questionnaire has been developed in collaboration with the Psychiatry Department to study the psychological impact of Beirut’s triple blow on the intended faculty development initiative (appendix 3). This 4-point Likert scale questionnaire is composed of three parts: the first part includes direct questions assessing the impacts of COVID-19, of the financial crisis and of the Beirut blast on the daily life of our trainees and of their loved ones; the second and third parts contain indirect questions assessing daily stress and detecting early features of depression. The psychological questionnaire was administered before starting the first session and at the end of the tenth training session to interpret the results of the assessment framework through the lens of Beirut’s triple blow’s psychological impact.

*Pilot study (validity evidence) of the different questionnaires:*

Two content experts were involved in developing and reviewing the questionnaires that were pilot tested prior to their implementation on a sample of five faculty members not enrolled in the FDP, making sure they are in line with outcomes being assessed. Physician Examiners and trainees were adequately trained prior to FDP administration and given specific guidelines about the questionnaires and rubrics in order to ensure the accuracy and the integrity of the data collected during the response process. The internal structure validity evidence was evaluated by two independent raters whose inter-rater reliability was evaluated by the kappa correlation coefficient that accounts for the random-chance occurrence of rater agreement (Kappa = 0.84). Reliability was evaluated using Cronbach’s alpha for internal consistency with a value of 0.89.

*Pre-Workshops Psychological Questionnaire:*

- The psychological questionnaire (appendix 3) was administered at the beginning of the FDP prior to workshop I on February 7, 2022, 18 months after the Beirut Blast, 28 months following the start of Lebanon’s economic meltdown, and 24 months after Lebanon’s first confirmed case of Covid-19.
- All questions whose answers mean was above the two-point cut-off (i.e.: Considerably, extremely, somewhat more than usual, much more than usual, more than half the days, nearly Every Day) according to the Likert scale were considered as answers indicating that their authors were significantly affected psychologically by Beirut’s triple blow.

Data was electronically collected via Microsoft Forms (©Microsoft 2022). SPSS version 22 (IBM Corp) was used to analyze the results. Wilcoxon Signed Rank Test was used to analyze results of the surveys administered following each workshop. Significance level was taken at p-value<0.05. This study was approved by the IRB committees of both SGUB (IRB-RES/O/002-22/0122) and Johns Hopkins University IRB (HIRB00014603) where the first author is currently enrolled in the Master of Education in the Health Professions (MEHP). All participants provided written informed consent prior to enrolment in the study.

## Results

There were 20 men (57%) and 15 women (43%) participants in the Faculty Development Program. The participants' average age was 49 years (range: 30 – 77) and their average job experience was 14 years (range: 1 – 46) ( Table 1).

**Table 1.** Study population demographics.

	Variable	Frequency	Percentage/Standard Deviation
Gender			
	Male	20	57%
	Female	15	43%
	Total	35	100%
Age			
	Mean	49	SD=11
	Min	30	
	Max	77	
Years of Experience			
	Mean	14	SD=11
	Min	1	
	Max	46	

Overall, 77% of all participants found the workshops to be of excellent quality with a mean value of overall satisfaction of the quality of the program of 4.1 ± 0.5 ( Table 3).

### Pre-workshop psychological questionnaire

All attendees were significantly affected by the Beirut triple blow with answers scoring two and above except for question 1: ‘In the past 30 days, how much were you bothered by the Aug 4, 2020 explosion: nightmares, fear, trouble concentrating, mood changes’ = 1.5, question 9: ‘During the past week, have you lost confidence in yourself’ = 1.6 and question 10: ‘During the past two weeks, have you felt little interest or pleasure in doing things or felt down, depressed, or hopeless’ = 1.7 ( Table 2).

**Table 2.** Pre-workshops psychological questionnaire results

	In the past 30 days, how much were you bothered by the Aug 4, 2020 explosion: nightmares, fear, trouble concentrating, mood changes?	In the past year, did you feel that your basic lifestyle had changed (education of children, access to healthcare, grocery shopping) due to financial needs?	How worried are you about getting COVID-19?	How worried are you about infecting your loved ones with COVID-19?	During the past week, have you been over worried?	During the past week, have you felt constantly under stress or strain?	During the past week, have you been able to enjoy your normal day-to-day activities?	During the past week, have you felt that you could not overcome your difficulties?	During the past week, have you lost confidence in yourself?	During the past two weeks, have you felt little interest or pleasure in doing things or felt down, depressed, or hopeless?
**Mean**	**1.5**	**2.5**	**2.1**	**2.8**	**2.3**	**2.3**	**2.0**	**2.1**	**1.6**	**1.7**
Minimum	1	1	1	1	1	1	1	1	1	1
Maximum	3	4	4	4	4	4	3	4	3	4

**Table 3.** Kirkpatrick’s level 1 (Satisfaction questionnaire) results

Instructor Assessment	Level of satisfaction (average of 10 modules, Mean ± SD)	Spearman rho’s
Academic skillfulness of the instructor	4.3 +/- 0.42	0.82*
Lecturing method and the ability to transfer the concepts to learners	4.1 +/- 0.45	0.82**
Ability of the instructor in class management	4.1 +/- 0.42	0.72**
Use of active teaching methods and engaging the learners	3.9 +/- 0.46	0.69**
Ability to respond to the inquiries and questions of learners	4.2 +/- 0.47	0.71**
Frequency of using practical examples during teaching	3.9 +/- 0.62	0.78**
Course content assessment	Level of satisfaction (average of 10 modules, Mean ± SD)	Spearman rho’s
Effectiveness of the contents of the course in increasing your knowledge	4.1 +/- 0.52	0.86**
Relationship between training course and your organizational needs as defined by the Faculty Affairs Committee	3.9 +/- 0.57	0.72**
Up-to-datedness of the contents of the course	4.2 +/- 0.47	0.92**
Quality of content in the training course	4.0 +/- 0.54	0.87**
Course support assessment	Level of satisfaction (average of 10 modules, Mean ± SD)	Spearman rho’s
Your satisfaction with the duration of the course	4.0 +/- 0.45	0.56**
Desirability of educational location and environment	3.7 +/- 0.63	0.62**
Quality of lighting in the classes	3.9 +/- 0.51	0.66**
Ventilation and adequacy of cooling/heating system	3.3 +/- 0.94	0.49**
Treatment of instructors toward you	4.4 +/- 0.44	0.78**
Overall satisfaction	Level of satisfaction (average of 10 modules, Mean ± SD)	Spearman rho’s
Satisfaction with the quality of workshops	4.1 +/- 0.47	-
Satisfaction with the way of conducting workshops	4.1 +/- 0.64	0.91**

*Kirkpatrick’s level 1 (Satisfaction questionnaire) results:*

- According to the results of the first stage of Kirkpatrick evaluation, the number of participants that said the workshops were of excellent quality was 27 (77%). Out of the 35 participants, 31 (90%) expressed complete satisfaction with the workshop format. In every other section of the questionnaire, more than 50 % of the respondents said the quality was very good.
- We computed the mean and SD for every item of the level I questionnaire for all workshops combined ( Table 3). On a scale of 1 to 5, instructor assessment averaged 4.1, course content assessment averaged 4.2, course support assessment averaged 3.9, while the overall assessment of the quality of the workshops averaged 4.1.
- Spearman correlation testing yielded a highly significant correlation (p=0.00<0.05) as evidenced by the high value of the R coefficient between the rank of each workshop in item “Satisfaction with the quality of workshop” and the rank of each workshop in the rest of the items ( Table 2).

*Kirkpatrick’s level 2 (Learning questionnaires) results:*

At the end of each module, participants were administered three questionnaires.

- The mean percentage of correct answers to the first questionnaire (that includes 10 to 15 multiple choice questions (MCQs) directly related to each workshop’s content (workshops 1 to 9, workshop 10 not included) thus directly testing post-session cognitive learning), was 76% ranging from 65% (workshop 3) to 88% (Workshop 2) (SD=7.7) ( Table 4)

**Table 4.** Kirkpatrick’s level 2 (MCQ Learning questionnaires) results.

Level II Results	(Mean % of Correct answers)	Standard Deviation (SD)
**Workshop 1**	79	SD=8.6
**Workshop 2**	88	SD=14
**Workshop 3**	65	SD=13
**Workshop 4**	70	SD=13
**Workshop 5**	66	SD=17
**Workshop 6**	84	SD=9.9
**Workshop 7**	77	SD= 12
**Workshop 8**	68	SD=17
**Workshop 9**	85	SD=11
**Workshop 10**	(Does not apply)	(Does not apply)
**Total Workshops**	76	7.7

- The mean result for the Retrospective Pre questionnaire of all workshops averaged 2.3 SD=0.57 (Scale 1 to 5) while the mean result for the Post questionnaire of all workshops increased to 3.6 SD=0.50 (scale 1 to 5) (p=0.00<0.05) ( Table 5). Moreover, for every workshop, the increase of the score between the Retrospective Pre and Post questionnaires was highly significant (p=0.00<0.05) ( Table 5).

**Table 5.** Kirkpatrick’s level 2 (RPP questionnaires) results.

	Retrospective Pre-Q	Post-Q	Wilcoxon Signed Rank Test
Workshop 1	2.5	3.3	Z=-3.9 p˂0.05
Workshop 2	1.7	4.1	Z=-3.9 p˂0.05
Workshop 3	2.8	3.8	Z=-4.1 p˂0.05
Workshop 4	1.7	3.8	Z=-4.1 p˂0.05
Workshop 5	2.9	3.4	Z=-4.0 p˂0.05
Workshop 6	1.9	4.1	Z=-4.1 p˂0.05
Workshop 7	1.9	3.8	Z=-3.9 p˂0.05
Workshop 8	2.3	2.9	Z=-3.2 p˂0.05
Workshop 9	1.9	3.4	Z=-3.4 p˂0.05
Workshop 10	Does not apply	Does not apply	Does not apply
Total Workshops	2.3	3.6	Z=-5.4 p˂0.05

*Post-Workshops Psychological Questionnaire results show there successful learning despite the economical and psycho-social challenges* ( Table 6).

**Table 6.** Post-Workshops Psychological Questionnaire results.

	In the past 30 days, how much were you bothered by the Aug 4, 2020 explosion: nightmares, fear, trouble concentrating, mood changes?	In the past year, did you feel that your basic lifestyle had changed (education of children, access to healthcare, grocery shopping) due to financial needs?	How worried are you about getting COVID-19?	How worried are you about infecting your loved ones with COVID-19?	During the past week, have you been over worried?	During the past week, have you felt constantly under stress or strain?	During the past week, have you been able to enjoy your normal day-to-day activities?	During the past week, have you felt that you could not overcome your difficulties?	During the past week, have you lost confidence in yourself?	During the past two weeks, have you felt little interest or pleasure in doing things or felt down, depressed, or hopeless?
**Mean**	**1.4**	**2.7**	**2.2**	**3.0**	**2.1**	**2.3**	**2.2**	**2.0**	**1.6**	**1.4**
Min	1	1	1	2	1	1	1	1	1	1
Max	3	4	4	4	3	4	4	4	3	4

*Comparison between Pre and Post workshops psychological questionnaires* show there is no statistically significant difference in the psychological status of the trainees as assessed by our psychological questionnaire prior to and after FDP administration, indicating that the effects of Beirut’s triple blow were still affecting most of our participants with the same intensity six months after having started the workshops ( Table 7). A majority of faculty (55%) were significantly affected by Beirut’s triple blow with a mean score of 2.1 SD=0.54 at the pre-FDP psychological questionnaire and a mean score of 2.1 SD=0.32 at the post-FDP psychological questionnaire.

**Table 7.** Comparison between pre and post workshops psychological questionnaires.

		In the past 30 days, how much were you bothered by the Aug 4, 2020 explosion: nightmares, fear, trouble concentrating, mood changes?	In the past year, did you feel that your basic lifestyle had changed (education of children, access to healthcare, grocery shopping) due to financial needs?	How worried are you about getting COVID- 19?	How worried are you about infecting your loved ones with COVID- 19?	During the past week, have you been over worried?	During the past week, have you felt constantly under stress or strain?	During the past week, have you been able to enjoy your normal day-to-day activities?	During the past week, have you felt that you could not overcome your difficulties?	During the past week, have you lost confidence in yourself?	During the past two weeks, have you felt little interest or pleasure in doing things or felt down, depressed, or hopeless?
**Pre-** **workshop**	**Mean**	**1.5**	**2.5**	**2.1**	**2.8**	**2.3**	**2.3**	**2.0**	**2.1**	**1.6**	**1.7**
	Min	1	1	1	1	1	1	1	1	1	1
	Max	3	4	4	4	4	4	3	4	3	4
**Post-** **Workshop**	**Mean**	1.4	2.7	2.2	3.0	2.1	2.3	2.2	2.0	1.6	1.4
	Min	1	1	1	2	1	1	1	1	1	1
	Max	3	4	4	4	3	4	4	4	3	4
**Wilcoxon ** **Signed ** **Rank Test**	Z	0.00	-1.9	-0.6	-1.0	-1.3	-0.55	-0.38	-1.6	0.00	-0.45
	Asymp. Sig. (2-tailed)	1.0	0.06	0.53	0.32	0.18	0.58	0.71	0.10	1.0	0.66

*Assessment framework results through the lens of Beirut’s triple blow’s psychological impact:*

We computed a variable entitled “Pre-Workshops Mean Psychological questionnaire” corresponding to the mean of the answers to the 10 questions of our psychological questionnaire.

*Kirkpatrick’s level I questionnaire:* a significant negative relationship (p˂0.05) between the score of the psychological questionnaire and the satisfaction with the quality of the workshops (p=0.00<0.05), and with the way of conducting the workshops (p=0.00<0.05). The more psychologically affected the participants are, the lower is their overall satisfaction with the workshops.

*Kirkpatrick’s Level II Questionnaire:*

- *MCQs results:*

Comparing the mean percentage of correct answers to all Level II workshops’ MCQs with the Pre-Workshops Mean Psychological questionnaire score yielded also a highly significant negative relationship (p=0.00<0.05). The more psychologically affected the participants are, the lower is their performance in answering the cognitive MCQs in all workshops. (p=0.00<0.05)

- *Self-assessment questionnaires (RPP):*

(1) The psychological status of the participants does not affect their Retrospective Pre-self-assessment regarding their potential performance in one of the workshops’ topics (p=0.35>0.05).

(2) The more psychologically affected the participants are, the lower is their self-assessment in the Post questionnaires of all workshops regarding their potential performance in one of the workshops’ topics (p=0.00<0.05). In other words, trainees that are less affected psychologically are more likely to benefit from the FDP.

## Discussion

These results demonstrate a significant improvement of the trainees’ self-perception and self-confidence as well as an enhancement of their ability to use the new teaching and learning methods after the intervention. Such programs have already proved their efficacy in improving teaching effectiveness in medical education under normal circumstances ^1– 7^. Unfortunately, SGUB FM’s launch stumbled upon simultaneous unprecedented circumstances that hit Lebanon’s capital, Beirut’s triple blow, mandating that these events be taken into consideration when evaluating the success of implementing a FDP.

Similar studies performed in the context of faculty development in medical education show satisfaction levels of 63 % ^13^, and a mean value of overall satisfaction for a whole FDP program of 3.6 ± 0.50 (Kim; 2015), and 4.2 ± 0.32 ^14^ on a 4-point Likert scale.

Coupled with a specifically created psychological questionnaire, the widely used Kirkpatrick’s evaluation framework was chosen for its simplicity and ease of application ^15^. We intentionally limited our assessment in this paper to Kirkpatrick’s Levels I and II to explore the degree to which our learners found the training favorable, engaging, and relevant to their jobs, as well as the degree to which they acquired the intended knowledge, skills, and confidence from the training. Results of both Levels III and IV will be interpreted, discussed, and diffused in a subsequent paper to be submitted next year, in 2023.

The effects of Beirut’s triple blow were still affecting most of our trainees with the same intensity six month after having started the workshops as evidenced by the absence of statistically significant difference between the results of the psychological questionnaire administered prior to and after the 10 workshops.

Results of level II (learning) show that the mean percentage of correct answers to the nine post-workshops MCQs is 76%, while the mean results for the RPP questionnaires of all workshops improved significantly from 2.3 SD=0.57 to 3.6 SD=0.50 (scale 1 to 5) (p<0.05). Heydari (2019) ^13^ has demonstrated a significant improvement in the scores of participants in the workshops before and after the intervention (p˂ 0.05), as well as Steinert (2016) ^7^ in a systematic review of faculty development initiatives designed to enhance teaching effectiveness have found 58% improvement in Level II pertaining to knowledge and skills. 

When interpreting the results of Kirkpatrick’s Levels I and II through the lens of the psychological questionnaire we have found that the more a trainee is psychologically affected with a high mean psychological score, the less his/her post-workshop satisfaction, and the less he/she performs as evidenced by a decrease in the score of the cognitive MCQs and of the Post questionnaire of the RPP framework.

It is well known that constant stress, as in the case of our faculty who have all witnessed Beirut’s triple blow, can lead to emotional problems, depression, panic attacks, or other forms of anxiety including post-traumatic stress disorder (PTSD) ^16^. In such circumstances, developing problem-focused and emotion-focused coping mechanisms are effective ways to manage stress ^17^. Moreover, positive reinterpretation and growth, planning, active coping, seeking social support for emotional reasons, acceptance, mental disengagement, and focusing on emotional venting are all crucial stress coping techniques on the individual level ^18^. Additionally, Iqbal (2011) ^19^ shows that top management and university administration should focus their attention on providing regular communication and support in order to improve their staff performance and well-being.

## Conclusion

The peculiarity of this study lies within its ability to highlight the effect of the psychological status of the trainees and how this affects their performance using the Kirkpatrick’s evaluation framework. It also underscores that significant learning can occur amidst stress and uncertainty at least in resilient trainees living in a country with a long-standing history of turmoil. 

## Data Availability

Figshare: Faculty Development, https://doi.org/10.6084/m9.figshare.21803820.v2 ^20^. This project contains the following underlying data: FD 1 TO 10 Final.sav Figshare: Faculty Development Appendices, https://doi.org/10.6084/m9.figshare.21975572.v1 ^21^. This project contains the following extended data: Faculty Development Appendices Data are available under the terms of the Creative Commons Attribution 4.0 International license (CC-BY 4.0).
