# Successful implementation of Medical Education Faculty Development Project at Saint George University of Beirut in the immediate post triple blow to Beirut

**DOI:** 10.12688/mep.19519.2

**Published:** 2023-11-02

**Authors:** Alexandre Nehme, Rachel Btaiche, Marc Jreij, Jizel Jahjah, George Karam, Anne Belcher

**Affiliations:** 1 Faculty of Medicine, Saint George University of Beirut, Ashrafiye, N/A, Lebanon; 2 School of Education, Johns Hopkins University, Baltimore, USA

**Keywords:** Faculty Development, Medical Education, Kirkpatrick’s evaluation framework, Beirut’s triple blow, University Medical Center

## Abstract

**Background:**

The aim of this study is to explore the efficacy of the Faculty Development Program (FDP) implemented at the Saint George University of Beirut-Faculty of Medicine (SGUB FM) under exceptional circumstances as the triple blow to Beirut.

**Methods:**

The Faculty Development, directed towards a cohort of 35 faculty members, is composed of two major components: methodology of teaching and techniques of assessment. The Kirkpatrick’s assessment model, in combination with a specifically designed psychological questionnaire, were chosen to assess the effectiveness of the faculty development initiative.

**Results:**

Results of the different questionnaires were interpreted individually, then through the lens of the psychological questionnaire. A majority of faculty (55%) were significantly affected psychologically by Beirut’s triple blow and 77% of all participants found the workshops to be of excellent quality (Kirkpatrick’s Level I). Moreover, Kirkpatrick’s level II results yielded a 76% mean percentage of correct answers to post-workshops MCQs and a significant improvement in the mean results of the self-assessment questionnaires, administered before and after each workshop. Results also show that the more a trainee is psychologically affected, the less he/she performs as evidenced by a decrease in the satisfaction rate as well as in the score of the cognitive MCQs and of the self-assessment questionnaires.

**Conclusions:**

This study was able to highlight that significant learning can occur amidst exceptional circumstances like the Beirut triple blow and administration should invest in professional growth to retain its faculty.

## Introduction

In the dynamic tapestry of global medical education, one constant remains: the necessity to evolve and adapt. Faculty development programs (FDPs) have stood as testaments to this evolution, with robust evidence from both the U.S. and the international community affirming their impact on amplifying teaching effectiveness ^1– 7^. The exigencies of the COVID-19 era further catalyzed this evolution, making the shift from traditional classrooms to innovative online platforms not just a trend, but an imperative, especially when in-person pedagogical interactions became untenable. Studies like Zuo *et al.*, 2021 ^8^ offer insights into this paradigm shift, while other research underscores the pivotal role of educators' psychological well-being in pedagogical success ^9, 10^.

Yet, how do these initiatives fare amidst unparalleled adversities? Most academic contexts have not been tested against a confluence of challenges as seen in Beirut. Here, the city grappled with a triad of crises - the destabilization of its banking system since 2019, the ravages of the COVID-19 pandemic, and the profound trauma of the Beirut Explosion on August 4, 2020. The repercussions were particularly acute for the venerable Saint George Hospital University Medical Center (SGHUMC), a beacon of medical excellence since 1878.

On the edge of these tumultuous events, SGHUMC was in the midst of transformative strides. The inauguration of the Saint George University of Beirut (SGUB) in 2018 marked the dawn of an avant-garde medical school (SGUB FM) with visions set on global accreditation and pioneering educational approaches. However, challenges were manifold, with the institution confronting the inertia of a two-decade-old didactic curriculum and a prolonged hiatus in faculty development.

Confronted by these layered challenges, the leadership of the university demonstrated an unwavering resolve towards modernization, envisioning a rejuvenated curriculum and bolstered faculty capabilities. Demonstrating an inspiring resilience against the myriad challenges Beirut presented, the faculty stood resolute in their pedagogical mission. This study explores the inception and challenges of the Curriculum Development Program at SGUB FM, emphasizing the relentless pursuit to empower educators amidst such monumental challenges.

## Methods

### Study population

In preparation for the inaugural MED I class in September 2022, SGUB FM’s Dean’s Office targeted a faculty development initiative at a group of 35 faculty members, none of whom had prior formal training in faculty development in medical education. This cohort encompassed members of the Curriculum Committee and educators responsible for the MED I and MED II curriculum segments, all of whom concurrently serve as practicing physicians at SGHUMC.

### Settings and study design

To support this initiative, an interventional study was launched, piloting a Faculty Development Program (FDP) with an emphasis on student/learner-centered pedagogy. This FDP was meticulously crafted to be interactive, positioning participants at the heart of all discussions. Beyond the core sessions, the workshops were enriched with self-paced readings and follow-up tasks. Upon completing the program, participants were awarded certificates.

***Program Modules:***

The FDP encompassed the following ten workshops:

1. Introduction & Characteristics of Adult Learning
2. Learner-centered Classes
3. Group Work
4. Interactive Lecturing
5. Project-based Learning
6. Flipped Learning
7. Assessment: Formative and Summative
8. Assessment: Rubrics
9. Team-based Learning
10. Reflections

***Implementation*:**

For each module, a facilitator from the American University of Beirut Center for Teaching and Learning was invited to the SGUB FM premises to lead an interactive workshop. These sessions spanned over a six-month period, with ten sessions in total, each lasting two hours.

***Program Components*:**

The FDP was designed around two primary components: teaching methodologies and assessment techniques. Given the importance of reflections in enhancing learning experiences, the program culminated with a dedicated session for participants to share and discuss any transformations in their teaching methodologies.

***Rationale for Workshop Selection*:**

Despite the COVID-19 pandemic, there were key reasons for choosing in-person workshops:

- Vaccination: All participants had two doses of the Pfizer vaccine, lowering transmission risk.
- Safety: On-site sessions followed strict safety rules, like mask-wearing, good ventilation, and distancing.
- Better Engagement: Face-to-face meetings enable richer discussions and real-time feedback compared to virtual settings.
- Avoiding Screen Burnout: Too much virtual meeting time can lead to fatigue. In-person sessions offered a screen break.
- Practical Learning: Some topics, like group tasks, thrive in a hands-on, in-person environment.

The Kirkpatrick’s (1976) ^11^ assessment model consisting of four levels (appendix 1), was adopted to evaluate the effectiveness of the faculty development initiative due to its simplicity, its assessment of a limited number of variables, the ease of its evaluation criteria, the lack of requirement to collect participants’ basic data or past performances as well as the independence of individual and environmental variables ^12^. Two levels were developed: Reaction – Level 1 detailed in appendix 2, and Learning – Level 2 detailed in sessions 1 to 9 ‘Post workshop Multiple Choice Questions’ and ‘Retrospective Pre and Post’ questionnaires.

The retrospective pre–post method (RPP) offers an alternative method to the traditional ‘pre-post design’ that usually relies on the stability of the participants standard of measurement for the dimension being assessed from one data point to the next ^13.^ As the learners' perception of the dimension(s) being measured evolves, they readjust the criteria for their self-rating: the response shift bias ^14, 15.^ When using the RPP method, the ratings of understanding before (referred to as the ‘retrospective pre’) and after (referred to as the ‘retrospective post’) the intervention employ the same metric because data are taken at the same point in time, i.e., at the conclusion of training, thus reducing such bias ^15.^ The behavior level (level III) and the results level (level IV) are evaluated by qualitative and quantitative data (open- and closed-ended questions) with trainee faculty after three months and nine months of workshops completion respectively **.** Results of both Levels III and IV will be interpreted, discussed and diffused in a subsequent paper to be submitted later.

Finally, a specific questionnaire has been developed in collaboration with the Psychiatry Department to study the psychological impact of Beirut’s triple blow on the intended faculty development initiative (appendix 3). This 4-point Likert scale questionnaire is composed of three parts: the first part includes direct questions assessing the impacts of COVID-19, of the financial crisis and of the Beirut blast on the daily life of our trainees and of their loved ones; the second and third parts contain indirect questions assessing daily stress and detecting early features of depression. The psychological questionnaire was administered before starting the first session and at the end of the tenth training session to interpret the results of the assessment framework through the lens of Beirut’s triple blow’s psychological impact.

***Pilot study (validity evidence) of the different questionnaires**:*

Two content experts were involved in developing and reviewing the questionnaires that were pilot tested prior to their implementation on a sample of five faculty members not enrolled in the FDP, making sure they are in line with outcomes being assessed. Physician Examiners that were in charge of course delivery and assessment and Enrolled Trainees were adequately trained prior to FDP administration and given specific guidelines about the questionnaires and rubrics in order to ensure the accuracy and the integrity of the data collected during the response process. The internal structure validity evidence was evaluated by two independent raters whose inter-rater reliability was evaluated by the kappa correlation coefficient that accounts for the random-chance occurrence of rater agreement (Kappa = 0.84). Reliability was evaluated using Cronbach’s alpha for internal consistency ^16^ with a value of 0.89.

***Pre-and Post-Workshops Psychological Questionnaire**:*

- The psychological questionnaire (appendix 3) was administered at the beginning of the FDP prior to workshop I on February 7, 2022, 18 months after the Beirut Blast, 28 months following the start of Lebanon’s economic meltdown, and 24 months after Lebanon’s first confirmed case of Covid-19.
- All questions whose answers mean was above the two-point cut-off (i.e.: [Considerably, extremely], [somewhat more than usual, much more than usual], [more than half the days, nearly Every Day]) according to the Likert scale were considered as answers indicating that their authors were significantly affected psychologically by Beirut’s triple blow.

Data was electronically collected via Microsoft Forms (©Microsoft 2022). SPSS version 22 (IBM Corp) was used to analyze the results. Wilcoxon Signed Rank Test was used to analyze results of the surveys administered following each workshop. The Wilcoxon Signed Rank Test was chosen over the t-test due to the non-normal and potentially ordinal nature of the psychological questionnaire data, which often arises from Likert-type scales. Additionally, the Wilcoxon test is more robust to outliers and aptly designed for paired samples, like our pre-post workshop measurements. Significance level was taken at p-value<0.05. This study was approved by the IRB committees of both SGUB (IRB-RES/O/002-22/0122) and Johns Hopkins University IRB (HIRB00014603) where the first author is currently enrolled in the Master of Education in the Health Professions (MEHP). All participants provided written informed consent prior to enrolment in the study.

## Results

There were 20 men (57%) and 15 women (43%) participants in the Faculty Development Program. The participants' average age was 49 years (range: 30 – 77) and their average job experience was 14 years (range: 1 – 46) ( Table 1).

**Table 1.** Study population demographics.

	Variable	Frequency	Percentage/Standard Deviation
Gender			
	Male	20	57%
	Female	15	43%
	Total	35	100%
Age			
	Mean	49	SD=11
	Min	30	
	Max	77	
Years of Experience			
	Mean	14	SD=11
	Min	1	
	Max	46	

Overall, 77% of all participants found the workshops to be of excellent quality with a mean value of overall satisfaction of the quality of the program of 4.1 ± 0.5 ( Table 3).

### Pre-workshop psychological questionnaire

Participants demonstrated significant psychological adverse effects from the Beirut triple catastrophe, as indicated by statistically significant findings (p < 0.05) in 7 out of 10 survey items. Responses for the following three items, however, did not reach significance:

1. 'In the past 30 days, how much were you affected by the Aug 4, 2020, explosion: nightmares, fear, trouble concentrating, mood changes?' (Mean score = 1.5)
2. 'During the past week, have you lost confidence in yourself?' (Mean score = 1.6)
3. 'Over the previous two weeks, have you experienced diminished interest or pleasure in activities, or felt down, depressed, or hopeless?' (Mean score = 1.7).

( Table 2).

**Table 2.** Pre-workshops psychological questionnaire results.

	In the past 30 days, how much were you bothered by the Aug 4, 2020 explosion: nightmares, fear, trouble concentrating, mood changes?	In the past year, did you feel that your basic lifestyle had changed (education of children, access to healthcare, grocery shopping) due to financial needs?	How worried are you about getting COVID-19?	How worried are you about infecting your loved ones with COVID-19?	During the past week, have you been over worried?	During the past week, have you felt constantly under stress or strain?	During the past week, have you been able to enjoy your normal day-to-day activities?	During the past week, have you felt that you could not overcome your difficulties?	During the past week, have you lost confidence in yourself?	During the past two weeks, have you felt little interest or pleasure in doing things or felt down, depressed, or hopeless?
**Mean**	**1.5**	**2.5**	**2.1**	**2.8**	**2.3**	**2.3**	**2.0**	**2.1**	**1.6**	**1.7**
Minimum	1	1	1	1	1	1	1	1	1	1
Maximum	3	4	4	4	4	4	3	4	3	4

**Table 3.** Kirkpatrick’s level 1 (Satisfaction questionnaire) results.

Instructor Assessment	Level of satisfaction (average of 10 modules, Mean ± SD)	Spearman rho’s
Academic skillfulness of the instructor	4.3 +/- 0.42	0.82*
Lecturing method and the ability to transfer the concepts to learners	4.1 +/- 0.45	0.82**
Ability of the instructor in class management	4.1 +/- 0.42	0.72**
Use of active teaching methods and engaging the learners	3.9 +/- 0.46	0.69**
Ability to respond to the inquiries and questions of learners	4.2 +/- 0.47	0.71**
Frequency of using practical examples during teaching	3.9 +/- 0.62	0.78**
Course content assessment	Level of satisfaction (average of 10 modules, Mean ± SD)	Spearman rho’s
Effectiveness of the contents of the course in increasing your knowledge	4.1 +/- 0.52	0.86**
Relationship between training course and your organizational needs as defined by the Faculty Affairs Committee	3.9 +/- 0.57	0.72**
Up-to-datedness of the contents of the course	4.2 +/- 0.47	0.92**
Quality of content in the training course	4.0 +/- 0.54	0.87**
Course support assessment	Level of satisfaction (average of 10 modules, Mean ± SD)	Spearman rho’s
Your satisfaction with the duration of the course	4.0 +/- 0.45	0.56**
Desirability of educational location and environment	3.7 +/- 0.63	0.62**
Quality of lighting in the classes	3.9 +/- 0.51	0.66**
Ventilation and adequacy of cooling/heating system	3.3 +/- 0.94	0.49**
Treatment of instructors toward you	4.4 +/- 0.44	0.78**
Overall satisfaction	Level of satisfaction (average of 10 modules, Mean ± SD)	Spearman rho’s
Satisfaction with the quality of workshops	4.1 +/- 0.47	-
Satisfaction with the way of conducting workshops	4.1 +/- 0.64	0.91**

*Kirkpatrick’s level 1 (Satisfaction questionnaire) results:*

- According to the results of the first stage of Kirkpatrick evaluation, the number of participants that said the workshops were of excellent quality was 27 (77%). Out of the 35 participants, 31 (90%) expressed complete satisfaction with the workshop format. In every other section of the questionnaire, more than 50 % of the respondents said the quality was very good.
- We computed the mean and SD for every item of the level I questionnaire for all workshops combined ( Table 3). On a scale of 1 to 5, instructor assessment averaged 4.1, course content assessment averaged 4.2, course support assessment averaged 3.9, while the overall assessment of the quality of the workshops averaged 4.1.
- Spearman correlation testing yielded a highly significant correlation (p=0.00<0.05) as evidenced by the high value of the R coefficient between the rank of each workshop in item “Satisfaction with the quality of workshop” and the rank of each workshop in the rest of the items ( Table 2).

*Kirkpatrick’s level 2 (Learning questionnaires) results:*

At the end of each module, participants were administered three questionnaires.

- The mean percentage of correct answers to the first questionnaire (that includes 10 to 15 multiple choice questions (MCQs) directly related to each workshop’s content (workshops 1 to 9, workshop 10 not included) thus directly testing post-session cognitive learning), was 76% ranging from 65% (workshop 3) to 88% (Workshop 2) (SD=7.7) ( Table 4)
- The mean result for the Retrospective Pre questionnaire of all workshops averaged 2.3 SD=0.57 (Scale 1 to 5) while the mean result for the Post questionnaire of all workshops increased to 3.6 SD=0.50 (scale 1 to 5) (p=<0.05) ( Table 5). Moreover, for every workshop, the increase of the score between the Retrospective Pre and Post questionnaires was significant (p=<0.05) ( Table 5).

**Table 4.** Kirkpatrick’s level 2 (MCQ Learning questionnaires) results.

Level II Results	(Mean % of Correct answers)	Standard Deviation (SD)
**Workshop 1**	79	SD=8.6
**Workshop 2**	88	SD=14
**Workshop 3**	65	SD=13
**Workshop 4**	70	SD=13
**Workshop 5**	66	SD=17
**Workshop 6**	84	SD=9.9
**Workshop 7**	77	SD= 12
**Workshop 8**	68	SD=17
**Workshop 9**	85	SD=11
**Workshop 10**	(Does not apply)	(Does not apply)
**Total Workshops**	76	7.7

**Table 5.** Kirkpatrick’s level 2 (RPP questionnaires) results.

	Retrospective Pre-Q	Post-Q	Wilcoxon Signed Rank Test
Workshop 1	2.5	3.3	Z=-3.9 p˂0.05
Workshop 2	1.7	4.1	Z=-3.9 p˂0.05
Workshop 3	2.8	3.8	Z=-4.1 p˂0.05
Workshop 4	1.7	3.8	Z=-4.1 p˂0.05
Workshop 5	2.9	3.4	Z=-4.0 p˂0.05
Workshop 6	1.9	4.1	Z=-4.1 p˂0.05
Workshop 7	1.9	3.8	Z=-3.9 p˂0.05
Workshop 8	2.3	2.9	Z=-3.2 p˂0.05
Workshop 9	1.9	3.4	Z=-3.4 p˂0.05
Workshop 10	Does not apply	Does not apply	Does not apply
Total Workshops	2.3	3.6	Z=-5.4 p˂0.05

*Post-Workshops Psychological Questionnaire results show there successful learning despite the economical and psycho-social challenges* ( Table 6).

**Table 6.** Post-Workshops Psychological Questionnaire results.

	In the past 30 days, how much were you bothered by the Aug 4, 2020 explosion: nightmares, fear, trouble concentrating, mood changes?	In the past year, did you feel that your basic lifestyle had changed (education of children, access to healthcare, grocery shopping) due to financial needs?	How worried are you about getting COVID-19?	How worried are you about infecting your loved ones with COVID-19?	During the past week, have you been over worried?	During the past week, have you felt constantly under stress or strain?	During the past week, have you been able to enjoy your normal day-to-day activities?	During the past week, have you felt that you could not overcome your difficulties?	During the past week, have you lost confidence in yourself?	During the past two weeks, have you felt little interest or pleasure in doing things or felt down, depressed, or hopeless?
**Mean**	**1.4**	**2.7**	**2.2**	**3.0**	**2.1**	**2.3**	**2.2**	**2.0**	**1.6**	**1.4**
Min	1	1	1	2	1	1	1	1	1	1
Max	3	4	4	4	3	4	4	4	3	4

*Comparison between Pre and Post workshops psychological questionnaires* show there is no statistically significant difference in the psychological status of the trainees as assessed by our psychological questionnaire prior to and after FDP administration, indicating that the effects of Beirut’s triple blow were still affecting most of our participants with the same intensity six months after having started the workshops ( Table 7). A majority of faculty (55%) were markedly affected by Beirut’s triple blow with a mean score of 2.1 SD=0.54 at the pre-FDP psychological questionnaire and a mean score of 2.1 SD=0.32 at the post-FDP psychological questionnaire.

**Table 7.** Comparison between pre and post workshops psychological questionnaires.

		In the past 30 days, how much were you bothered by the Aug 4, 2020 explosion: nightmares, fear, trouble concentrating, mood changes?	In the past year, did you feel that your basic lifestyle had changed (education of children, access to healthcare, grocery shopping) due to financial needs?	How worried are you about getting COVID-19?	How worried are you about infecting your loved ones with COVID-19?	During the past week, have you been over worried?	During the past week, have you felt constantly under stress or strain?	During the past week, have you been able to enjoy your normal day-to-day activities?	During the past week, have you felt that you could not overcome your difficulties?	During the past week, have you lost confidence in yourself?	During the past two weeks, have you felt little interest or pleasure in doing things or felt down, depressed, or hopeless?
**Pre-** **workshop**	**Mean**	**1.5**	**2.5**	**2.1**	**2.8**	**2.3**	**2.3**	**2.0**	**2.1**	**1.6**	**1.7**
	Min	1	1	1	1	1	1	1	1	1	1
	Max	3	4	4	4	4	4	3	4	3	4
**Post-** **Workshop**	**Mean**	1.4	2.7	2.2	3.0	2.1	2.3	2.2	2.0	1.6	1.4
	Min	1	1	1	2	1	1	1	1	1	1
	Max	3	4	4	4	3	4	4	4	3	4
**Wilcoxon** **Signed** **Rank Test**	Z	0.00	-1.9	-0.6	-1.0	-1.3	-0.55	-0.38	-1.6	0.00	-0.45
	Asymp. Sig. (2-tailed)	1.0	0.06	0.53	0.32	0.18	0.58	0.71	0.10	1.0	0.66

*Assessment framework results through the lens of Beirut’s triple blow’s psychological impact:*

We computed a variable entitled “Pre-Workshops Mean Psychological questionnaire” corresponding to the mean of the answers to the 10 questions of our psychological questionnaire.

- *Kirkpatrick’s level I questionnaire:* a significant negative relationship (p˂0.05) between the score of the psychological questionnaire and the satisfaction with the quality of the workshops (p=0.00<0.05), and with the way of conducting the workshops (p=0.00<0.05). The more psychologically affected the participants are, the lower is their overall satisfaction with the workshops.
- *Kirkpatrick’s Level II Questionnaire:*
- *MCQs results:*
- Comparing the mean percentage of correct answers to all Level II workshops’ MCQs with the Pre-Workshops Mean Psychological questionnaire score yielded also a highly significant negative relationship (p=0.00<0.05). The more psychologically affected the participants are, the lower is their performance in answering the cognitive MCQs in all workshops. (p=0.00<0.05)
- *Self-assessment questionnaires (RPP):*
- (1) The psychological status of the participants does not affect their Retrospective Pre-self-assessment regarding their potential performance in one of the workshops’ topics (p=0.35>0.05).
- (2) The more psychologically affected the participants are, the lower is their self-assessment in the Post questionnaires of all workshops regarding their potential performance in one of the workshops’ topics (p=0.00<0.05). In other words, trainees that are less affected psychologically are more likely to benefit from the FDP.

## Discussion

This study showed a marked boost in trainees' self-awareness, confidence, and their adeptness to new instructional strategies post-intervention. These faculty development programs are known to enhance teaching effectiveness in standard medical education settings ^1– 7^.

These programs remain a prominent topic in medical literature, designed to enhance the skills and knowledge of faculty members, subsequently improving the overall quality of medical education ^1– 7^. They come in various formats, from brief workshops and seminars to comprehensive long-term degree courses. Their content encompasses a wide range of subjects, including curriculum design, instructional strategies, assessment strategies, leadership, research methods, and the integration of technology. In terms of duration, some are concise, lasting just a few days, while others can extend over several years. The COVID-19 pandemic has seen many of these programs transition to online platforms ^8^, although some have adopted a blended approach ^17^. Regardless of the format or content, continuous evaluation and feedback are crucial components, ensuring the programs remain effective and up to date.

The initiation of SGUB FM's program occurred amidst a backdrop of significant upheaval in Beirut, marked by socio-political turmoil, economic downturn, and the devastating 2020 port explosion. This unique context not only sets Beirut's medical faculty's challenges apart from their global counterparts but also underscores their resilience. Indeed, these unparalleled events in Beirut lend additional weight and significance to the outcomes of our study.

**Limitations of the Study:** While this study offers valuable insights, it's crucial to acknowledge its limitations. Firstly, the sample size was relatively small, limiting the generalizability of the findings. Additionally, the study primarily relied on self-reported data, which may introduce response bias. The external circumstances, including Beirut's triple blow, created a unique context that might not be fully replicable in other settings. Furthermore, the evaluation was limited to Kirkpatrick's Levels I and II, and future studies should explore Levels III and IV to provide a more comprehensive assessment of the program's impact.

Similar studies conducted in the realm of faculty development in medical education have shown satisfaction levels of 63% ^18^. The mean value of overall satisfaction for a whole FDP program ranged from 3.6 ± 0.50 (Kim, 2015) ^19^ to 4.2 ± 0.32 on a 4-point Likert scale. To assess the impact of our FDP, we employed a specifically designed psychological questionnaire in conjunction with Kirkpatrick's evaluation framework, chosen for its simplicity and ease of application ^12^. 

It's worth noting that the effects of Beirut's triple blow continued to impact most of our trainees with the same intensity even six months after commencing the workshops. This is evident in the absence of a statistically significant difference between the results of the psychological questionnaire administered before and after the 10 workshops.

Level II results (learning) indicate that the mean percentage of correct answers to the nine post-workshop MCQs is 76%. Additionally, the mean results for the RPP questionnaires of all workshops improved significantly from 2.3 (SD=0.57) to 3.6 (SD=0.50) on a 5-point scale (p<0.05). Previous studies, such as Heydari (2019) ^18^ and Steinert (2016) ^7^, have also demonstrated significant improvement in Level II pertaining to knowledge and skills.

When interpreting the results of Kirkpatrick's Levels I and II alongside the psychological questionnaire, a notable pattern emerges. Trainees experiencing higher psychological distress, as indicated by a high mean psychological score, tend to exhibit lower post-workshop satisfaction and reduced performance, as evidenced by decreased scores in cognitive MCQs and the Post questionnaire of the RPP framework.

**Implications for Future Faculty Development Initiatives:** These findings hold significant implications for shaping future faculty development initiatives. Firstly, they underscore the importance of considering contextual factors and the psychological well-being of trainees when designing and implementing such programs. Faculty development initiatives should incorporate strategies to address trainees' emotional well-being, particularly in challenging or crisis-prone environments.

Additionally, the study's results will inform the design and assessment of future faculty development initiatives. Understanding the impact of training on Kirkpatrick's Levels I and II provides a foundation for evaluating effectiveness. Future research should delve into Levels III and IV, focusing on behavioral change and organizational impact, to provide a more comprehensive view of program outcomes.

Moreover, given the unique context of Beirut's triple blow and its lasting effects on trainees, future initiatives should include proactive measures to support faculty members' mental and emotional well-being. Proven strategies such as stress coping mechanisms, emotional support, and regular communication from top management should be integrated into program planning ^20– 22^.

## Conclusion

In conclusion, while recognizing the study's limitations, these findings will serve as a valuable guide for developing more resilient and effective faculty development programs that can thrive even in challenging circumstances. The study's distinctiveness lies in its ability to illuminate the interplay between trainees' psychological status and their performance, set against the backdrop of Beirut's crises, as assessed by the Kirkpatrick’s evaluation framework. Furthermore, it underscores the potential for significant learning amidst stress and uncertainty, particularly in resilient trainees embedded in a country familiar with turmoil. Drawing upon global practices and contextualizing them to Beirut's unique challenges, the lessons learned from this study will contribute significantly to the continuous enhancement of faculty development initiatives worldwide.

## Data Availability

Figshare: Faculty Development, https://doi.org/10.6084/m9.figshare.21803820.v2 ^23^. This project contains the following underlying data: FD 1 TO 10 Final.sav Figshare: Faculty Development Appendices, https://doi.org/10.6084/m9.figshare.21975572.v1 ^24^. This project contains the following extended data: Faculty Development Appendices Data are available under the terms of the Creative Commons Attribution 4.0 International license (CC-BY 4.0).
