# Supplementary material for: Successful implementation of Medical Education Faculty Development Project at Saint George University of Beirut in the immediate post triple blow to Beirut
Source: MedEdPublish (2016). 2023 Nov 2;13:12. Originally published 2023 Feb 28. [Version 2] doi: 10.12688/mep.19519.2 (PMC10835100; doi:10.12688/mep.19519.2)
Supplement: Peer review history

# Reviewer report: Reviewer response for version 2

**DOI:** 10.21956/mep.21345.r35218

**Published:** 2024-02-01

**Authors:** Toni Ungaretti

**Affiliations:** 1 School of Education, Johns Hopkins, Baltimore, MD, USA

**Successful implementation of Medical Education Faculty Development Project at Saint George University of Beirut in the immediate post triple blow to Beirut**

 Nehme A, Btaiche R, Jreij M, Jahjah J, Karam G and Belcher A

Comments

 This is a review of the revised manuscript. The manuscript presents the examination of the impact of an innovative faculty development program that was implemented after the experience of the destabilization of the banking system, the COVID-19 pandemic, and the Beirut Explosion in 2020. The revised iteration of the manuscript provides additional detail and clarification that address identified needs in the original submission. It more effectively reflects the authors’ work and attention to detail that is indicated in the study outcome. It makes a contribution to global faculty development initiatives.

Introduction

 The revised introduction both captures and focuses attention on the importance of this issue. The citations of faculty development studies and especially those in disruption provide a stronger context for the study. Additional explanation and citations focused on psychological factors strengthen the context for the focus of this study. The authors might want to include detailed descriptions of the severe external disruptions related to the facilities, persons, processes, and procedures to set the foundation for the importance of this study. This coupled with the effort to seek accreditation and transform faculty development make this study unique in its contribution to knowledge. The authors might want to consider that descriptions such as “venerable”, “…a beacon of medical excellence since 1878”, and Avante-garde be provided with additional detail to give context (important to this work) to their meaning.

Methods

 Study population

 Demographics such as early, mid, and late career stage would be a useful addition as a lens to review the data. Information on this would help determine if there is a need to differentiate the curriculum based on experience.

 Settings and Study Design

 This section is much improved as it includes important details that add strength to the study and a clearer picture to guide replication. An example of a typical session would provide further insight into the structure of the intervention. It would illustrate the emphasis on the two primary components, teaching methodologies and assessment techniques. In Program Modules, the breakdown of the methods into the specific sessions with headings provides clear organization. Given that the reflection workshop is the emphasis of the last module, it would help the readers to have an explanation of how this skill is woven through the various sessions.   The use of trained facilitators is commendable and addresses issues related to teaching approaches.

 The inclusion of the Psychiatry Department provides an interprofessional collaborative model that can inform future work of similar studies. The use of the retrospective pre-post method is an interesting choice with a literature base to support its use for this type of study.  The authors effectively use Kirkpatrick’s Evaluation Model and note that future research will address Levels III and IV of this model. It is a challenge to show change at levels III and IV, so the second paper will be a welcome addition to the literature. The inclusion of a pilot study strengthens the impact of this work. Additional information to support the validation of the questionnaires is included. Steps have been taken to check for validity.

Results

 The results section is organized and detailed. The tables clearly convey the results. Limiting the use of the term “significant” to indicate p-value would eliminate any confusion. The authors might want to report the results under Kirkpatrick’s level I as “27/31 (77%)” to make it clearer to the reader.  

Discussion

 The Discussion presents clearer details and relates them more effectively to the introduction and the findings with accompanying citations to extend the impact of this work. This revision strengthens the study and will be a guide to future researchers. The authors should be commended for the extensive discussion on limitations and their inclusion of research related to this work. The implications of this study follow from the introduction and the findings.

Conclusion

 The conclusion aligns this effort with global faculty development initiatives and research agendas.

Have any limitations of the research been acknowledged?

No

Is the work clearly and accurately presented and does it cite the current literature?

Yes

If applicable, is the statistical analysis and its interpretation appropriate?

Partly

Are all the source data underlying the results available to ensure full reproducibility?

Yes

Is the study design appropriate and is the work technically sound?

Yes

Are the conclusions drawn adequately supported by the results?

Yes

Are sufficient details of methods and analysis provided to allow replication by others?

Partly

Reviewer Expertise:

Qualitative, quantitative, and mixed methods

I confirm that I have read this submission and believe that I have an appropriate level of expertise to confirm that it is of an acceptable scientific standard.

# Reviewer report: Reviewer response for version 2

**DOI:** 10.21956/mep.21345.r35219

**Published:** 2023-11-24

**Authors:** Ardi Findyartini

**Affiliations:** 1 Universitas Indonesia, Depok, West Java, Indonesia

I would like to highlight that I am fine with the revision.

Have any limitations of the research been acknowledged?

No

Is the work clearly and accurately presented and does it cite the current literature?

Partly

If applicable, is the statistical analysis and its interpretation appropriate?

I cannot comment. A qualified statistician is required.

Are all the source data underlying the results available to ensure full reproducibility?

Yes

Is the study design appropriate and is the work technically sound?

Partly

Are the conclusions drawn adequately supported by the results?

Partly

Are sufficient details of methods and analysis provided to allow replication by others?

Partly

Reviewer Expertise:

NA

I confirm that I have read this submission and believe that I have an appropriate level of expertise to confirm that it is of an acceptable scientific standard.

# Reviewer report: Reviewer response for version 1

**DOI:** 10.21956/mep.20912.r33394

**Published:** 2023-06-27

**Authors:** Ardi Findyartini

**Affiliations:** 1 Universitas Indonesia, Depok, West Java, Indonesia

The article titled ‘Successful implementation of Medical Education Faculty Development Project at Saint George University of Beirut in the immediate post triple blow to Beirut’ elaborates a significant attempt to provide adequate support through faculty development program (mainly workshops) for the faculty in the medical school in challenging circumstances. While such an effort is highly appreciated, I find this article is quite hard to follow and I cannot recommend further publication until it is substantially revised.

Introduction:

 The current introduction explained the context of the medical school without highlighting current literature on faculty development efforts in different medical schools around the world during the challenging circumstances (including COVID-19, limited resources, etc). It is recommended that the authors also explain the scope of faculty development better by incorporating key literature on faculty development (e.g. Steinert *et al.*, 2014, Steinert *et al.*, 2017 - BEME Guide no 40, etc), to later on place the current faculty development initiative better. The authors have used relevant literature in the discussion section, but did not elaborate on them well in the introduction. The introduction should also add the literature on the influence of psychological condition of the faculty towards the effectiveness of faculty development. Overall, the current introduction is still lacking a systematic analysis of the gap in the literature on faculty development in specific circumstances and rationale on why this study hence is important for readers. A clearer research question is also warranted.

Methods:

 The study setting as elaborated in the current introduction can be considered to be moved in the study setting in the methods section. The authors should elaborate on the medical school curriculum context in which the faculty enact their roles as teachers, as well as the faculty development program available before the present intervention is in place.

 The use of Kirkpatrick’s framework is appropriate. The instruments to measure the satisfaction and knowledge of the workshop participants were fairly explained.

 The description of the intervention should be more detailed. It is not adequate to just explain that the intervention is workshop, with selected topics. How was the workshop done? Who lead the workshop? What was the method for the workshop? Were there any activities following the workshops, etc. The authors might consider underlining the rationale of selecting workshops as a strategy in the current faculty development program. In other words, the explanation on validity of the tools being used in this study should be added with the ‘validity’ or trustworthiness of the workshops, hence the authors may provide both evidence on the effectiveness of the workshop and how and why the workshops were done.

Results:

 The results are quite compelling and well described. With further requirement to revise the introduction and methods section, it is sometimes quite challenging to grasp the meaning of the results.

Discussion:

 The authors have elaborated the main findings and provided reasoning using relevant literature. The discussion section however still requires further work. I would recommend the authors do not only discuss the level of satisfaction and knowledge, yet how the faculty development programs in the different literature were completed and in what circumstances. Given that the authors aim to highlight the special circumstances of faculty development program they have conducted in Beirut, discussing the contexts of different studies and how they are relevant with this present study is critical. Herewith, it is expected that the authors may elaborate further recommendations or impacts of this study in others settings, as well as specify the limitation of the present study better.

Conclusion:

 The conclusion is fair. Yet, clearer research questions/evaluation questions elaborated in the introduction should be able to strengthen the conclusion.

Have any limitations of the research been acknowledged?

No

Is the work clearly and accurately presented and does it cite the current literature?

Partly

If applicable, is the statistical analysis and its interpretation appropriate?

I cannot comment. A qualified statistician is required.

Are all the source data underlying the results available to ensure full reproducibility?

Yes

Is the study design appropriate and is the work technically sound?

Partly

Are the conclusions drawn adequately supported by the results?

Partly

Are sufficient details of methods and analysis provided to allow replication by others?

Partly

Reviewer Expertise:

NA

I confirm that I have read this submission and believe that I have an appropriate level of expertise to confirm that it is of an acceptable scientific standard, however I have significant reservations, as outlined above.

# Response

**Published:** 2023-09-28

**Authors:** Marc Jreij

**Affiliations:** Saint George Hospital University Medical Center, Lebanon

Dear Dr. Ungaretti,  

 I would like to express my gratitude for your thorough review and constructive feedback on our manuscript submitted to MedEdPublish. Your insights have been invaluable in refining our paper. Please find below a summary of the changes made in response to your comments:

 Introduction:

 1. The opening has been revised for a more compelling introduction.

 2. The consideration of including the psychological impact on faculty in the aim has been incorporated.

 Methods: Study Population: No changes were required as the description was deemed adequate.

 Settings and Study Design:

 1. Clarification has been provided for the first sentence, explaining the underlined phrase regarding the Faculty Development Program (FDP).

 2. Additional details on the instruments and their intended purpose have been incorporated for improved clarity.

 3. The application of Kirkpatrick's Levels 1 and 2 remains unchanged due to your affirmation. 4. Consistency has been ensured in terms used to describe different roles. Definitions and distinctions have been provided between terms like content experts, Physician Examiners, faculty, and trainees.

 5. We have retained the description of the data collection and analysis process for the Pre-Workshop Psychological Questionnaires, as they were clear and had IRB approval.

 Results:

 1. The presentation of results in tables remains unchanged.

 2. Statements of results have been clarified, and the term 'significantly' has been rephrased as suggested.

 3. Rationale for the application of the Wilcoxon Signed Rank Test over a t-test for the pre-post workshop psychological questionnaire results has been provided.

 4. MCQ results and Self-assessment questionnaires have been updated to report the p-value and the term “highly significant” has been omitted.

 Discussion:

 1. The last paragraph has been revised to better align with the findings and to ensure a clear connection with the core results of the study.

 2. We've identified and included the limitations of the study.

 3. A section has been added on how these results will be leveraged to shape future faculty development initiatives.

 In addition to your feedback, we have also integrated suggestions from other reviewers to produce a more polished and robust manuscript. I believe that these revisions have substantially improved the quality and clarity of our work. Once again, I appreciate your time and expertise in reviewing our manuscript. Looking forward to your continued support and feedback.  

 Warm regards,

# Response

**Published:** 2023-09-28

**Authors:** Marc Jreij

**Affiliations:** Saint George Hospital University Medical Center, Lebanon

Dear Dr. Findyartini,

 I sincerely appreciate the time and effort you have invested in reviewing our manuscript submitted to MedEdPublish. Your detailed feedback has provided us with a roadmap for essential revisions to enhance the quality and clarity of our work. Here's a summary of the modifications made in response to your feedback:

 Introduction:

 1. The introduction has been revised to incorporate a more in-depth exploration of current literature on faculty development efforts in various medical schools worldwide, particularly during challenging times such as the COVID-19 pandemic and resource limitations.

 2. An elaboration on the psychological condition of faculty and its influence on the effectiveness of faculty development has been included.

 3. A systematic analysis of the gap in the literature concerning faculty development in specific situations has been added to underline the significance and rationale of our study.

 4. The research question has been clarified and emphasized to provide readers with a clear direction of our study's focus.

 Methods:

 1. As suggested, we further elaborated on the medical school curriculum context.

 2. We've provided a detailed description of the pre-existing faculty development programs before introducing our initiative.

 3. A comprehensive breakdown of the intervention, specifically the workshops, has been provided. This includes information about how the workshop was conducted, who led it, its methodology, and post-workshop activities. Further, we elucidated the rationale behind selecting workshops as our faculty development strategy.

 4. The validity of the tools and the trustworthiness of the workshops have been expanded upon, providing a solid foundation for our research methodology. Results: While you found the results to be compelling, we have made slight modifications to ensure consistency and clarity, especially given the changes in the introduction and methods sections.

 Discussion:

 1. We have incorporated a more in-depth discussion of the contexts of various faculty development programs found in the literature, emphasizing the circumstances under which they were conducted.

 2. Drawing parallels between our study and others in terms of context, we've highlighted the uniqueness of our faculty development program in Beirut.

 3. Based on your feedback, we've provided more detailed recommendations and implications of our study for other settings and refined the limitations section for clarity.

 Conclusion: The conclusion has been restructured.

 In addition to your insightful comments, we've also taken into account suggestions from other reviewers. Collectively, these changes have significantly elevated the overall quality and impact of our manuscript. Again, thank you for your constructive feedback. I hope that our revisions meet your expectations and I look forward to hearing your thoughts on the modified manuscript.

# Reviewer report: Reviewer response for version 1

**DOI:** 10.21956/mep.20912.r32980

**Published:** 2023-03-30

**Authors:** Toni Ungaretti

**Affiliations:** 1 School of Education, Johns Hopkins, Baltimore, MD, USA

**Comments:**

 This study examines faculty development outcomes under disruptive situations and contributes a unique perspective to this discourse. A few adjustments would strengthen this work. Consider having the manuscript edited to communicate this work more effectively to readers. 

**Introduction:**

 The opening sentence needs to capture the attention of the reader. The first two sentences in the third paragraph of the Introduction can be used to create a compelling introduction: “Most faculty development initiatives have been tested in different settings, whether in the United States or abroad, and have proved their efficacy in improving teaching effectiveness in medical education ^1– 7^; however, such initiatives have still not yet been studied when applied in a combination of exceptional circumstances”.

 This entire second paragraph makes a much stronger opening paragraph for this paper.

 Is there an interest in including in the aim the psychological impact on the faculty in keeping with the psychological measures used with the faculty?

**Methods:**

**Study Population:**

 The description is adequate.

**Settings and Study Design:**

- The first sentence is not clear. Explain what is meant by the underlined phrase: “This is an interventional study where the proposed pilot Faculty Development Program (FDP), is designed under the auspices of student/learner-centered classes with the following modules…”.
- The study includes a clear and thorough explanation (supported by citations) plus an accurate application of the retrospective pre-post method.
- Additional information on the instruments and their intent would make this easier for the reader to follow.
- The application of Kirkpatrick’s Levels 1 and 2 is accurate and clear.
- Validity and reliability procedures are included. The questionnaires (including the psychological questionnaires) were developed by two content experts and piloted by five faculty.

*Pilot Study:*

- The terms used to describe different roles need to be consistent. It is not clear whether the terms *content experts *and *Physician Examiners* refer to the same group of people. The same issue applies to the terms *faculty* and *trainees*.
- The psychometrics on validity and reliability are reported.

*Pre-workshops Psychological Questionnaires*:

- The Likert scale indicators should be clearly discrete.
- The description of the data collection and analysis process for the Pre-Workshop Psychological Questionnaires is clear. IRB approval was obtained.

**Results:**

- Tables clearly display results.
- Provide clearer statements of results. It might help to replace the adjective ‘significantly’ to avoid confusion about the significance of the findings. An example is in the first sentence of the results of the pre-workshop psychological questionnaire. Instead, report that 7 of 10 questions were significant at p <0.05.
- Kirkpatrick’s Level I (Satisfaction questionnaire) and Level II (Learning questionnaires) results are clearly presented.
- Provide the rationale for using the Wilcoxon Signed Rank Test rather than a t-test to compare the pre-post workshop psychological questionnaire results.
- Interpretations of the results are more appropriate in the Discussion rather than in the Results section.
- MCQ results and Self-assessment questionnaires - Report the p-value and omit  “highly significant.” The comparison of these is a critical component of this study

**Discussion:**

- Explain how the last paragraph of the discussion section relates to the findings as it appears to introduce additional variables.
- Limitations of the study need to be identified.
- How will these results be used to inform future faculty development initiatives?

**References:**

- References are accurate and appropriate.

Have any limitations of the research been acknowledged?

No

Is the work clearly and accurately presented and does it cite the current literature?

Yes

If applicable, is the statistical analysis and its interpretation appropriate?

Partly

Are all the source data underlying the results available to ensure full reproducibility?

Yes

Is the study design appropriate and is the work technically sound?

Yes

Are the conclusions drawn adequately supported by the results?

Yes

Are sufficient details of methods and analysis provided to allow replication by others?

Partly

Reviewer Expertise:

Qualitative, quantitative, and mixed methods

I confirm that I have read this submission and believe that I have an appropriate level of expertise to confirm that it is of an acceptable scientific standard, however I have significant reservations, as outlined above.
